# Supplementary material for: An innovative approach using CRISPR-ribonucleoprotein packaged in virus-like particles to generate genetically engineered mouse models
Source: Nat Commun. 2025 Apr 11;16:3451. doi: 10.1038/s41467-025-58364-7 (PMC11992299; doi:10.1038/s41467-025-58364-7)
Supplement: Supplementary file 1 — Supplementary Information [file 41467_2025_58364_MOESM1_ESM.pdf]

Supplementary Information

An innovative approach using CRISPR-ribonucleoprotein packaged in virus-like particles to generate genetically engineered mouse models

Supplementary Fig. 1 Gene editing efficiencies at human or mouse targets using varying volumes of SpCas9/sgRNA or ABE8e/sgRNA packaged in VLPs in cell lines.

Supplementary Fig. 2 Comparison of embryo editing efficiency using SpCas9/sgRNA RNP alone versus SpCas9/sgRNA RNP packaged in VLPs targeting the *Gata3* gene in mouse embryos.

Supplementary Fig. 3 Mutation patterns in newborn mice generated via the CRISPR-VIM method.

Supplementary Fig. 4 Off-target effects in *Plin1* and *Tyr* mutant mice generated via CRISPR-VIM method.

Supplementary Fig. 5 Gene editing efficiency and Cas9 protein quantification of codon-optimized VLPs in Neuro-2a cells.

Supplementary Fig. 6 Mutation rate distributions in embryos edited via the CRISPR-VIM method.

Supplementary Fig. 7 Validation of transduction efficiency of AAV6 and DJ serotypes in mouse Neuro-2a cells, mESCs, and embryos through GFP expression.

Supplementary Fig. 8 Supplementary Fig. 8 Knock-in or indel mutation efficiencies using the CRISPR-VIM-based HDR strategy in mouse Neuro-2a cells and mESCs.

Supplementary Fig. 9 Embryonic development rates following the application of CRISPR-VIM method.

Supplementary Fig. 10 Quantification of VLPs for all experimental targets using P30 ELISA.

Supplementary Table 1. Sequences of single guide RNAs targeting human and mouse genomes.

Supplementary Table 2. Primer sequences for on-target gene editing analysis.

Supplementary Table 3. Sequences of off-target candidate sites for *Tyr* and *Plin1*.

Supplementary Table 4. Primer sequences for analyzing off-target effects of *Tyr* and *Plin1*.

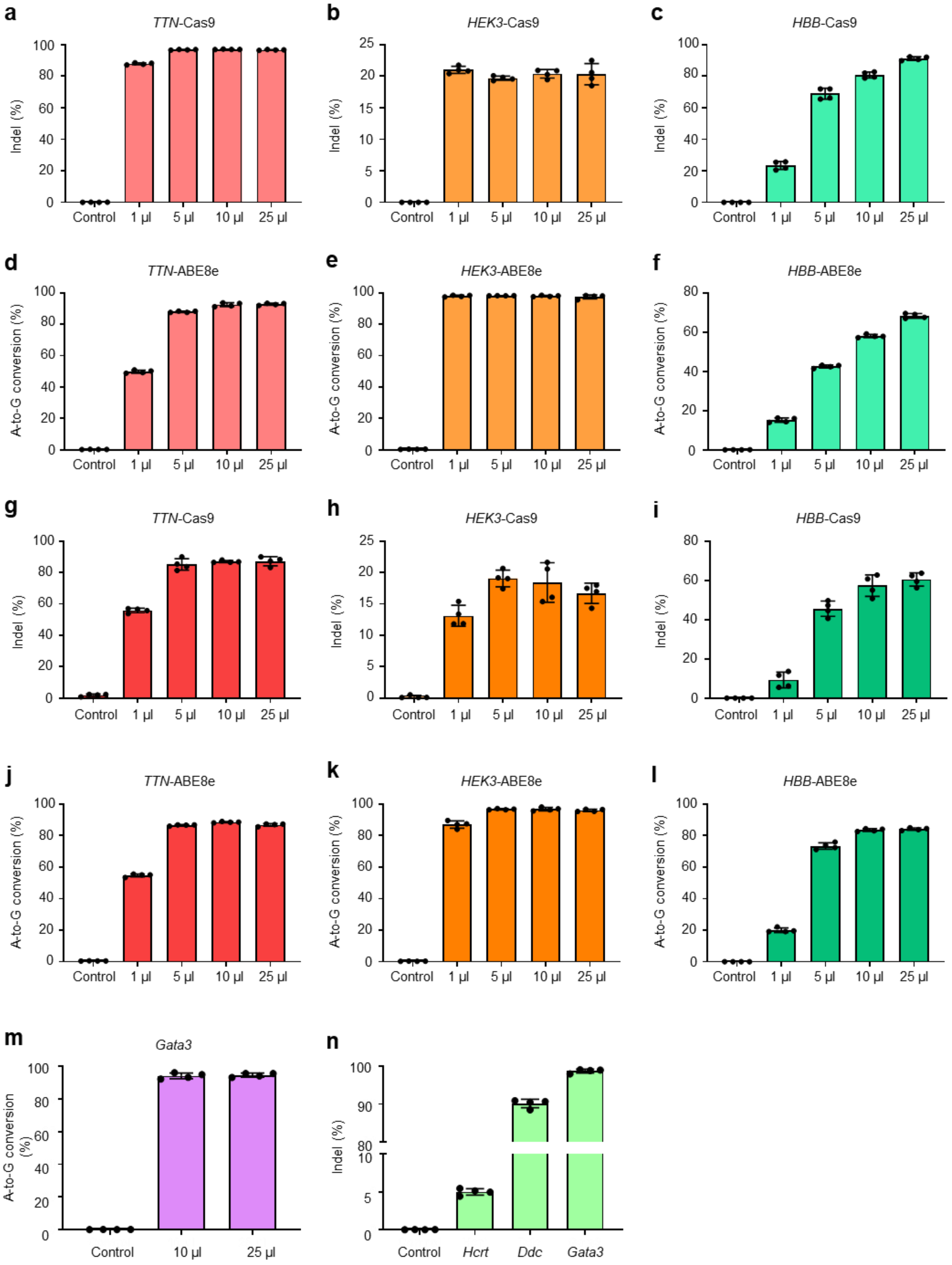
a
TTN-Cas9
Indel (%)
Control
1 µl
5 µl
10 µl
25 µl
b
HEK3-Cas9
c
HBB-Cas9
d
TTN-ABE8e
A-to-G conversion (%)
e
HEK3-ABE8e
f
HBB-ABE8e
g
h
i
j
k
l
m
Gata3
n
Hcrt
Ddc
Gata3

Supplementary Fig. 1 Gene editing efficiencies at human or mouse targets using varying volumes of SpCas9/sgRNA or ABE8e/sgRNA packaged in VLPs in cell lines. a-c, g-i, Indel frequencies at *TTN*, *HEK3*, and *HBB* targets in (**a-c**) HEK293T or (**g-i**) ARPE19 cells, based on the treatment volume of SpCas9/sgRNA packaged in VLPs. **d-f, j-l,** A-to-G conversion efficiencies using ABE8e/sgRNA packaged in VLPs in two human cell lines: (**d-f**) HEK293T or (**j-l**) ARPE19 cells (n = 4 for all conditions). **m,** A-to-G conversion frequencies at the *Gata3* locus in mESCs treated with 10 or 25 μl volumes of ABE8e/sgRNA packaged in VLPs (n = 4 for all conditions). **n,** Analysis of indel mutations at *Hcrt*, *Ddc*, and *Gata3* genes in mESCs treated with SpCas9/sgRNA packaged in VLPs (n = 4 for all conditions). Source data are provided as a Source Data file.

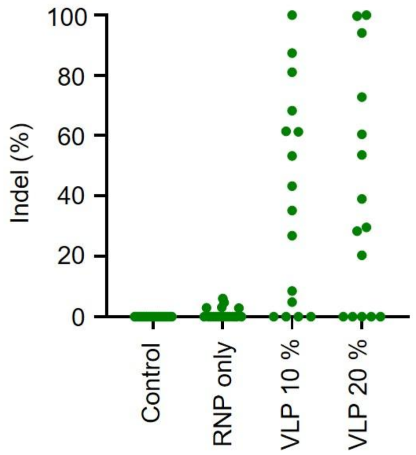

Supplementary Fig. 2 Comparison of embryo editing efficiency using SpCas9/sgRNA RNP alone versus SpCas9/sgRNA RNP packaged in VLPs targeting the *Gata3* gene in mouse embryos.

Evaluation of gene editing efficiency for the Gata3 gene in mouse embryos, comparing the performance of SpCas9/sgRNA RNP delivered alone versus RNP packaged in VLP. Source data are provided as a Source Data file.

a

Plin1 10%		Frequency (%)
WT	TCTGTGTGCAATGCCTATGAGAAGGGTGTACAGGGTGCCAGCAAC	
#1M	TCTGTGTGCAATGCCTATGAGAAGGGTGGTACAGGGTGCCAGCAAC	14.3
	TCTGTGTGCAATGCCTATGAGAAGG--GTACAGGGTGCCAGCAAC	13.9
#5F	TCTGTGTGCAATGCCTATGAGA---------AGGGTGCCAGCAAC	2.7
#6M	TCTGTGTGCAATGCCTATGAGAAGG--GTACAGGGTGCCAGCAAC	2.0
#10M	TCTGTGTGCAATGCCTATGAGA---------AGGGTGCCAGCAAC	1.4
#11M	TCTGTGTGCAATGCCTATGAGA---------AGGGTGCCAGCAAC	7.1
	TCTGTGTGCAATGCCTATGAGAAGGGT-TACAGGGTGCCAGCAAC	6.0
#16M	TCTGTGTGCAATGCCTATGAGA---------AGGGTGCCAGCAAC	10.5
#21F	TCTGTGTGCAATGCCTATGAGAAGGGTG--CAGGGTGCCAGCAAC	6.7
#32M	TCTGTGTGCAATGCCTATGAGAAGGT---ACAGGGTGCCAGCAAC	11.8
#42M	TCTGTGTGCAATGCCTATGAGA---------AGGGTGCCAGCAAC	26.0
	TCTGTGTGCAATGCCTATGAGAAGGGTGTTACAGGGTGCCAGCAAC	8.4
#48F	TCTGTGTGCAATGCCTATGAGA---------AGGGTGCCAGCAAC	1.6
#54M	TCTGTGTGCAATGCCTATGA-------TTACAGGGTGCCAGCAAC	19.4
#66M	TCTGTGTGCAATGCCTATGAGAAGGGT-----GGGTGCCAGCAAC	1.8

b

Plin1 20%		Frequency (%)
WT	TCTGTGTGCAATGCCTATGAGAAGGGTGTACAGGGTGCCAGCAAC	
#23F	TCTGTGTGCAATGCCTATGAGA---------AGGGTGCCAGCAAC	6.0
#31M	TCTGTGTGCAATGCCTATGAGA---------AGGGTGCCAGCAAC	21.8
	TCTGTGTGCAATGCCT---------------AGGGTGCCAGCAAC	16.5
#41F	TCTGTGTGCAATGCCTATGAGA---------AGGGTGCCAGCAAC	15.1
	TCTGTGTGCAATGCCTATGAGAAGGGTGGTACAGGGTGCCAGCAAC	6.5
	TCTGTGTGCAATGCCTATGAGA------TACAGGGTGCCAGCAAC	2.9
#47M	TCTGTGTGCAATGCCTATGAGA---------AGGGTGCCAGCAAC	22.0
#52M	TCTGTGTGCAATGCC-------------TACAGGGTGCCAGCAAC	4.7
#53M	TC-----------------------------AGGGTGCCAGCAAC	44.7
#63F	TCTGTGTGCAATGCCTATGAGAAGG--GTACAGGGTGCCAGCAAC	23.1

c

Tyr 20%		Frequency (%)
WT	AATGCACCTATCGGCCATAACAGAGACTCTTACATGGTTCCTTTCATACCG	
#2M	AATGCACCTATCGGCCGTGGCGGAGACTCTTACATGGTTCCTTTCATACCG	42.4
#3F	AATGCACCTGTCGGCCGTGGCGGAGACTCTTACATGGTTCCTTTCATACCG	15.0
	AATGCACCTATCGGCCGTGGCGGAGACTCTTACATGGTTCCTTTCATACCG	14.9

Supplementary Fig. 3 Mutation patterns in newborn mice generated via the CRISPR-VIM method. a, b, Mutation patterns in pups from mouse zygotes treated with (a) 10% or (b) 20% SpCas9/sgRNA packaged in VLPs targeting *Plin1*. **c,** Mutation patterns of newborn mice with the *Tyr* H420R induced by ABE8e/sgRNA packaged in VLPs. Red letters indicate mutation sites, green letters represent PAM sequences, and the underline indicates the target spacer sequence. The blue letter indicates substitution target A of the H420R mutation at the *Tyr* locus.

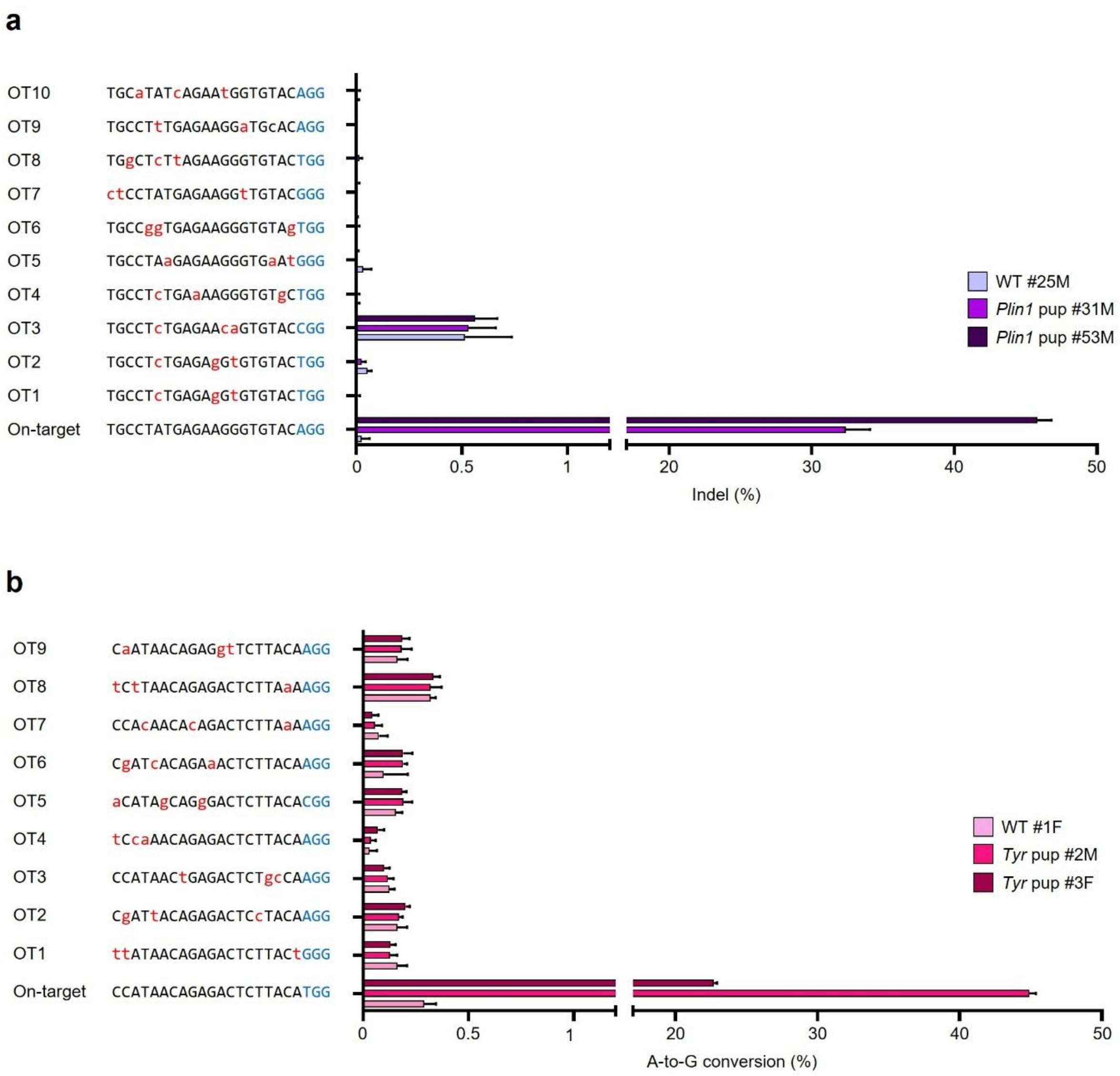

Supplementary Fig. 4 Off-target effects in *Plin1* and *Tyr* mutant mice generated via CRISPR-VIM method. a, b, Off-target effects analyzed by next-generation sequencing in *Plin1*- or *Tyr*-mutant mice. Source data are provided as a Source Data file.

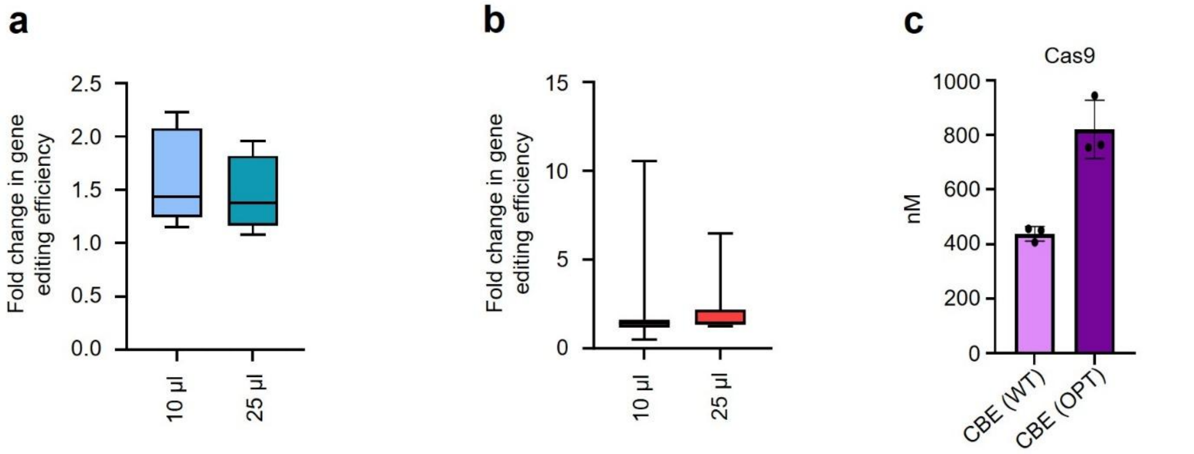

Supplementary Fig. 5 Gene editing efficiency and Cas9 protein quantification of codon-optimized VLPs in Neuro-2a cells. a, Fold changes in C-to-T conversion efficiency in human targets. **b,** Fold changes in C-to-T conversion efficiency at mouse target genes. **c,** Cas9 protein levels in VLPs packaged with CBE (WT) and codon-optimized VLPs packaged with CBE (OPT) measured by anti-FLAG or anti MLV(p30) ELISA (n = 3 for condition). Source data are provided as a Source Data file.

a

		Number of edited embryos (%)							Total embryos
Treatment time		< 1%	1~5 %	5~10 %	10~20 %	20~30%	30~40 %	> 40%	
0 h		8 (100.0)	0 (0.0)	0 (0.0)	0 (0.0)	0 (0.0)	0 (0.0)	0 (0.0)	8
1 h		54 (96.4)	1 (1.8)	1 (1.8)	0 (0.0)	0 (0.0)	0 (0.0)	0 (0.0)	56
5 h	*Tyr*-ABE	42 (85.7)	1 (2.0)	2 (4.1)	3 (6.1)	1 (2.0)	0 (0.0)	0 (0.0)	49
10 h		29 (78.4)	4 (10.8)	3 (8.1)	1 (2.7)	0 (0.0)	0 (0.0)	0 (0.0)	37
20 h		18 (43.9)	6 (14.6)	8 (19.5)	6 (14.6)	1 (2.4)	1 (2.4)	1 (2.4)	41

b

		Number of edited embryos (%)							Total embryos
Treatment time		< 1%	1~5 %	5~10 %	10~20 %	20~30%	30~40 %	> 40%	
0 h		4 (100.0)	0 (0.0)	0 (0.0)	0 (0.0)	0 (0.0)	0 (0.0)	0 (0.0)	4
1 h		35 (94.6)	0 (0.0)	0 (0.0)	0 (0.0)	2 (5.4)	0 (0.0)	0 (0.0)	37
5 h	*Plin1*-ABE	25 (62.5)	6 (15.0)	4 (10.0)	3 (7.5)	1 (2.5)	0 (0.0)	1 (2.5)	40
10 h		19 (55.9)	7 (20.6)	4 (11.8)	3 (8.8)	0 (0.0)	1 (2.9)	0 (0.0)	34
20 h		12 (25.5)	3 (6.4)	8 (17.0)	8 (17.0)	5 (10.6)	3 (6.4)	8 (17.0)	47

c

		Number of edited embryos (%)							Total embryos
VLP treatment %		< 1%	1~5 %	5~10 %	10~20 %	20~30%	30~40 %	> 40%	
10%	*Plin1*-ABE	7 (41.2)	1 (5.9)	4 (23.5)	2 (11.8)	0 (0.0)	1 (5.9)	2 (11.8)	17
20%		1 (5.0)	0 (0.0)	2 (10.0)	3 (15.0)	5 (25.0)	3 (15.0)	6 (30.0)	20

d

		Number of edited embryos (%)							Total embryos
VLP treatment %		< 1%	1~5 %	5~10 %	10~20 %	20~30%	30~40 %	> 40%	
10%	*Dnmt1*-ABE	18 (60.0)	6 (20.0)	2 (6.7)	2 (6.7)	0 (0.0)	2 (6.7)	0 (0.0)	30
20%		7 (29.2)	4 (16.7)	2 (8.3)	3 (12.5)	4 (16.7)	2 (8.3)	2 (8.3)	24

e

		Number of edited embryos (%)							Total embryos
VLP treatment %		< 1%	1~5 %	5~10 %	10~20 %	20~30%	30~40 %	> 40%	
10%	*Gata3*-ABE	18 (29.5)	5 (8.2)	5 (8.2)	7 (11.5)	4 (6.6)	4 (6.6)	18 (29.5)	61
20%		7 (10.4)	6 (9)	7 (10.4)	12 (17.9)	8 (11.9)	5 (7.5)	22 (32.8)	67

f

		Number of edited pups (%)							Total pups
VLP treatment %		< 1%	1~5 %	5~10 %	10~20 %	20~30%	30~40 %	> 40%	
10%	*Plin1*-Cas	31 (88.6)	1 (2.9)	1 (2.9)	2 (5.7)	0 (0.0)	0 (0.0)	0 (0.0)	35
20%		24 (88.9)	1 (3.7)	2 (7.4)	0 (0.0)	0 (0.0)	0 (0.0)	0 (0.0)	27

g

VLP treatment (+: 10%, ++: 20%)				Number of edited embryos (%)							Total embryos
Sperm	Fertilization	Culture		< 1%	1~5 %	5~10 %	10~20 %	20~30%	30~40 %	> 40%	
-	-	-		20 (100.0)	0 (0.0)	0 (0.0)	0 (0.0)	0 (0.0)	0 (0.0)	0 (0.0)	20
-	-	+		3 (20.0)	1 (6.7)	3 (20.0)	3 (20.0)	2 (13.3)	3 (20.0)	0 (0.0)	15
-	-	++		2 (16.7)	1 (8.3)	1 (8.3)	2 (16.7)	1 (8.3)	2 (16.7)	3 (25.0)	12
+	+	-	*Gata3*-ABE	5 (83.3)	0 (0.0)	0 (0.0)	1 (16.7)	0 (0.0)	0 (0.0)	0 (0.0)	6
+	+	+		1 (11.1)	2 (22.2)	1 (11.1)	3 (33.3)	0 (0.0)	1 (11.1)	1 (11.1)	9
++	++	-		2 (40.0)	0 (0.0)	1 (20.0)	2 (40.0)	0 (0.0)	0 (0.0)	0 (0.0)	5
++	++	++		2 (40.0)	0 (0.0)	2 (40.0)	1 (20.0)	0 (0.0)	0 (0.0)	0 (0.0)	5

h

VLP treatment (+: 10%, ++: 20%)				Number of edited embryos (%)							Total embryos
Sperm	Fertilization	Culture		< 1%	1~5 %	5~10 %	10~20 %	20~30%	30~40 %	> 40%	
-	-	-		24 (100.0)	0 (0.0)	0 (0.0)	0 (0.0)	0 (0.0)	0 (0.0)	0 (0.0)	24
-	-	+		1 (3.8)	2 (7.7)	5 (19.2)	3 (11.5)	5 (19.2)	4 (15.4)	6 (23.1)	26
-	-	++		7 (31.8)	3 (13.6)	0 (0.0)	2 (9.1)	1 (4.5)	2 (9.1)	7 (31.8)	22
+	+	-	*Plin1*-ABE	20 (83.3)	2 (8.3)	2 (8.3)	0 (0.0)	0 (0.0)	0 (0.0)	0 (0.0)	24
+	+	+		3 (23.1)	2 (15.4)	2 (15.4)	1 (7.7)	2 (15.4)	1 (7.7)	2 (15.4)	13
++	++	-		6 (66.7)	1 (11.1)	1 (11.1)	0 (0.0)	1 (11.1)	0 (0.0)	0 (0.0)	9
++	++	++		0 (0.0)	0 (0.0)	0 (0.0)	2 (40.0)	1 (20.0)	1 (20.0)	1 (20.0)	5

i

		Number of edited pups (%)							Total pups
VLP treatment %		< 1%	1~5 %	5~10 %	10~20 %	20~30%	30~40 %	> 40%	
10%	*Tyr-Ex4*-ABE	1 (14.3)	0 (0.0)	2 (28.6)	3 (42.9)	1 (14.3)	0 (0.0)	0 (0.0)	7
20%		57 (89.1)	1 (1.6)	1 (1.6)	0 (0)	3 (4.7)	0 (0.0)	2 (3.1)	8

j

		Edited embryo number (%)							Total embryos
VLP treatment %		< 1%	1~5 %	5~10 %	10~20 %	20~30%	30~40 %	> 40%	
10%	*Dnmt1*-CBE	46 (92.0)	2 (4.0)	2 (4.0)	0 (0.0)	0 (0.0)	0 (0.0)	0 (0.0)	50
20%		31 (96.9)	0 (0.0)	1 (3.1)	0 (0.0)	0 (0.0)	0 (0.0)	0 (0.0)	32

k

		Edited embryo number (%)							Total embryos
VLP treatment %		< 1%	1~5 %	5~10 %	10~20 %	20~30%	30~40 %	> 40%	
10%	*Hpd*-CBE	31 (88.6)	1 (2.9)	1 (2.9)	2 (5.7)	0 (0.0)	0 (0.0)	0 (0.0)	35
20%		24 (88.9)	1 (3.7)	2 (7.4)	0 (0.0)	0 (0.0)	0 (0.0)	0 (0.0)	27

Supplementary Fig. 6 Mutation rate distributions in embryos edited via the CRISPR-VIM method. a, b, Mutation rate distributions in edited embryos at **(a)** *Tyr* and **(b)** *Plin1* targets, depending on treatment duration (0, 1, 5, 10, and 20 h) with ABE8e/sgRNA in VLPs. **c, d, e,** Mutation rate distributions in edited embryos at **(c)** *Plin1*, **(d)** *Dnmt1*, and **(e)** *Gata3* targets, depending on treatment rates (10% or 20%) with ABE8e/sgRNA in VLPs. **f,** Mutation rate distributions in edited pups at the *Plin1* target, depending on treatment rates (10% or 20%) with SpCas9/sgRNA in VLPs. **g, h,** Mutation rate distributions in edited embryos at **(g)** *Gata3* and **(h)** *Plin1* targets, depending on treatment rates (10% or 20%) with ABE8e/sgRNA in VLPs during IVF process. **i,** Mutation rate distributions in mice born by treatment rates (10% or 20%) with ABE8e/sgRNA in VLPs targeting *Tyr*-Ex4 after IVF. **j, k,** Mutation rate distributions in edited embryos using human codon-optimized VLPs with AncBE4max/sgRNA targeting **(J)** *Dnmt1* or **(k)** *Hpd*, depending on the treatment rates (10% or 20%).

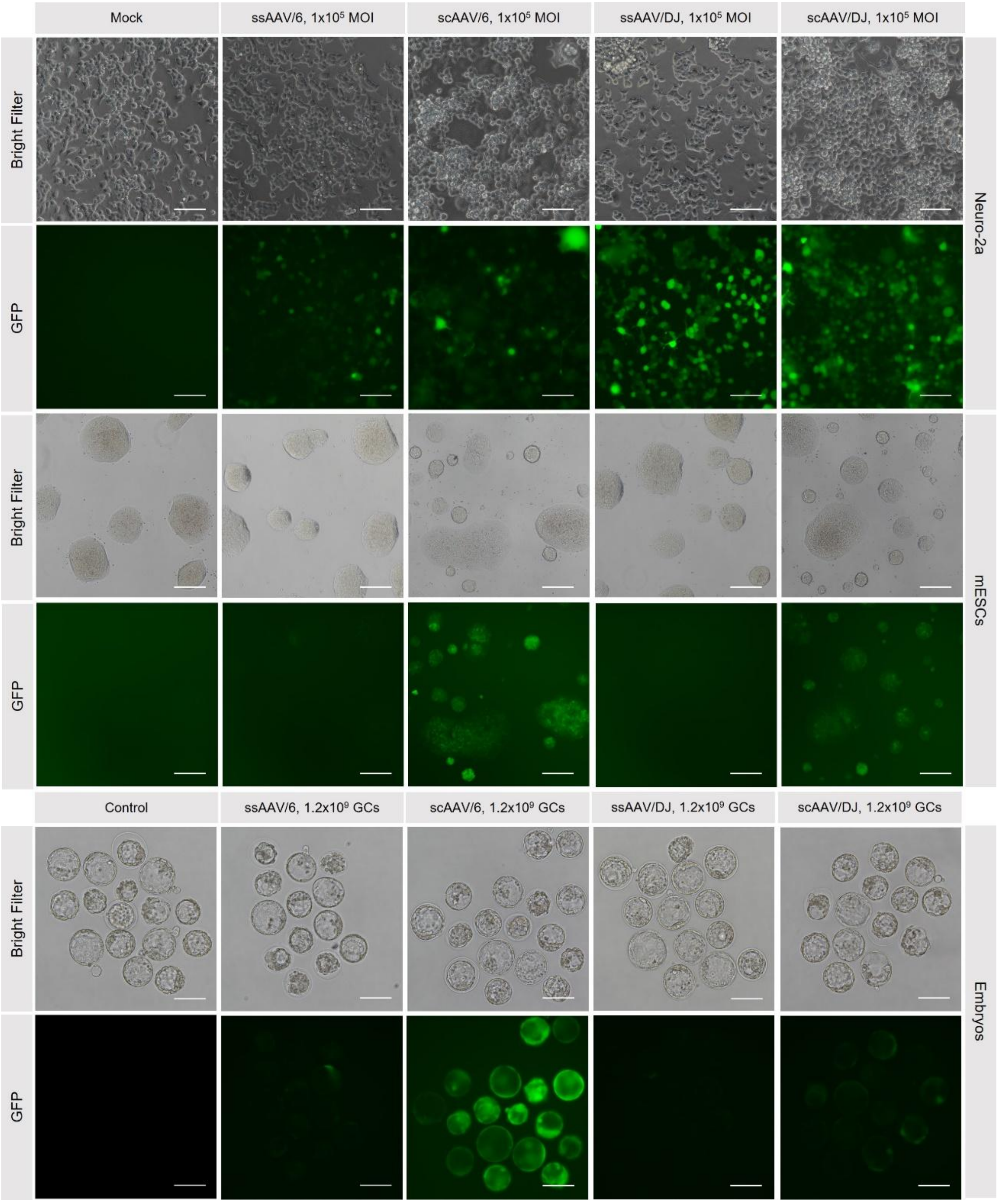

Supplementary Fig. 7 Validation of transduction efficiency of AAV6 and DJ serotypes in mouse Neuro-2a cells, mESCs, and embryos through GFP expression. Transduction efficiency of single-stranded AAV (ssAAV) and self-complementary AAV (scAAV) based on GFP expression levels in mouse Neuro-2a cells (Scale bars: 200 μm), mESCs (Scale bars: 100 μm), and embryos (Scale bars: 100 μm) across AAV/6 and AAV/DJ serotypes. Source data are provided as a Source Data file.

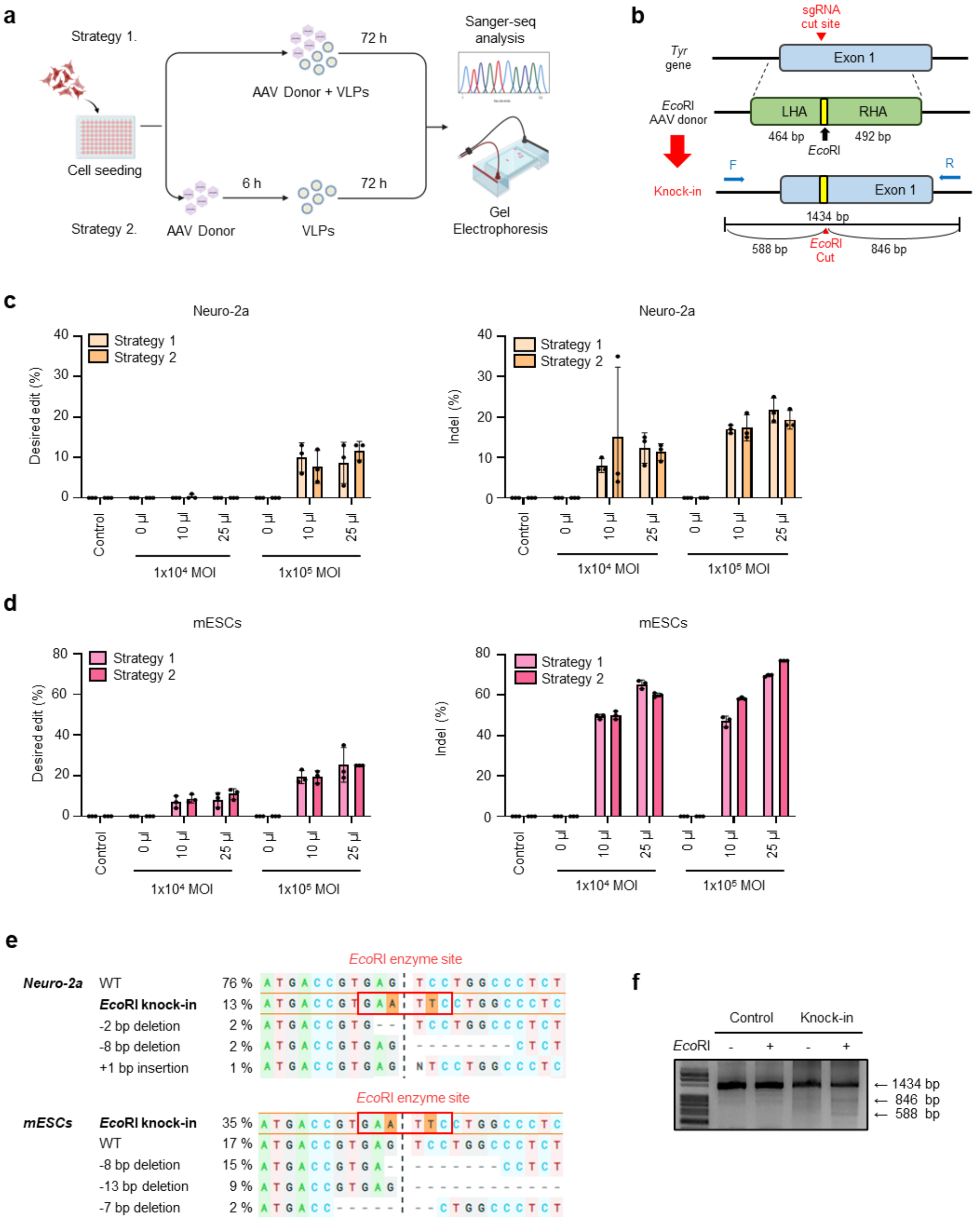

Supplementary Fig. 8 Knock-in or indel mutation efficiencies using the CRISPR-VIM-based HDR strategy in mouse Neuro-2a cells and mESCs. a, Schematic of the CRISPR-VIM-based HDR strategy using AAV-based donors. In Strategy 1, AAV donors and VLPs are added simultaneously the day after cell seeding. In Strategy 2, the AAV donors are added first and incubated for 6 hours, followed by VLP addition. Created in BioRender. Kim, K. (2025) https://BioRender.com/m78p943. **b,** Strategy

to insert the *EcoRI* enzyme sequence into exon 1 of the *Tyr* gene. LHA: left homology arm; RHA: right homology arm. **c, d,** Comparison of knock-in and indel mutation efficiencies between two CRISPR-VIM-mediated HDR strategies targeting *Tyr* in mouse Neuro-2a cells and mESCs (n = 3 for all condition). **e,** *EcoRI* knock-in efficiency analyzed by Sanger sequencing. **f,** Verification of *EcoRI* knock-in efficiency in mESCs by in vitro cleavage assay. Source data are provided as a Source Data file.

a

VLP treatment		No. of examined embryos	No. of two-cell stage embryos (%)	No. of blastocysts (%)	No. of transferred embryos	No. of offspring (%)	Mutant ratio (%)	
	Target						No. of desired mutants/total blastocyst (%)	No. of desired mutants/total offspring (%)
0%	-	362	224 (62)	167 (75)	-	-	0/46 (0)	-
10%	*Plin1* -ABE8e	50	21 (42)	17 (81)	-	-	10/17 (59)	-
	Dnmt1 -ABE8e	50	42 (84)	33 (79)	-	-	12/30 (40)	-
	Gata3 -ABE8e	98	70 (71)	65 (93)	-	-	43/71 (61)	-
	Plin1 -Cas9	594	445 (75)	-	445	68 (15)	-	12/68 (18)
	Dnmt1 -CBE	35	28 (80)	24 (86)	-	-		-
	Hpd -CBE	98	70 (71)	35 (50)	-	-		-
20%	*Plin1* -ABE8e	50	28 (56)	20 (71)	-	-	19/20 (95)	-
	Dnmt1 -ABE8e	51	33 (65)	24 (73)	-	-	17/24 (71)	-
	Gata3 -ABE8e	103	86 (83)	67 (78)	-	-	60/67 (90)	-
	Plin1 -Cas9	548	322 (59)	-	322	64 (20)	-	7/64 (11)
	Dnmt1 -CBE	35	28 (80)	20 (71)	-	-		-
	Hpd -CBE	98	66 (67)	27 (40)	-	-		-

b

VLP treatment				No. of examined embryos	No. of two-cell stage embryos (%)	No. of blastocysts (%)	No. of transferred embryos	No. of offspring (%)	Mutant ratio (%)	
Target	Sperm	IVF	Culture						No. of desired mutants/total blastocyst (%)	No. of desired mutants/total offspring (%)
Control	-	-	-	101	74 (73)	39 (53)	-	-	0/33 (0)	-
Gata3-ABE8e	-	-	+	40	32 (80)	18 (56)	-	-	12/18 (67)	-
	-	-	++	39	28 (72)	12 (43)	-	-	10/12 (83)	-
	+	+	-	52	23 (44)	6 (26)	-	-	1/6 (17)	-
	+	+	+	51	25 (49)	9 (36)	-	-	8/9 (89)	-
	++	++	-	46	16 (35)	6 (38)	-	-	3/5 (60)	-
	++	++	++	45	13 (29)	5 (38)	-	-	3/5 (60)	-
Plin1-ABE8e	-	-	+	55	45 (82)	26 (58)	-	-	25/26 (96)	-
	-	-	++	59	38 (64)	22 (58)	-	-	15/22 (68)	-
	+	+	-	87	54 (62)	24 (44)	-	-	4/24 (17)	-
	+	+	+	88	53 (60)	13 (25)	-	-	10/13 (77)	-
	++	++	-	85	38 (45)	9 (24)	-	-	3/9 (33)	-
	++	++	++	87	37 (43)	5 (14)	-	-	5/5 (100)	-
Tyr-ABE8e	-	-	+	30	19 (63)	11 (58)	-	-	6/7 (86)	-
	-	-	++	32	13 (41)	8 (62)	-	-	5/8 (63)	-
	-	-	++	95	46 (48)	-	46	9 (20)	-	2/9 (22)

c

VLP treatment		No. of examined embryos	No. of two-cell stage embryos (%)	No. of blastocysts (%)	No. of transferred embryos	No. of offspring (%)	Mutant ratio (%)	
	Target						No. of desired mutants/total blastocyst (%)	No. of desired mutants/total offspring (%)
0%	-	33	22 (67)	17 (77)	-	-	0	-
5%	*Kcnq4*	737	501 (68)	8/70 (11)	390	12 (3)	1/8 (13)	1/12 (8)

d

VLP treatment		No. of examined embryos	No. of two-cell stage embryos (%)	No. of blastocysts (%)	No. of transferred embryos	No. of offspring (%)	Mutant ratio (%)	
	Target						No. of desired mutants/total blastocyst (%)	No. of desired mutants/total offspring (%)
	Control	81	36 (44)	31 (86)	-	-	0 (0)	-
Combi	*Plin1*+*Kcnq4*-Cas	99	63 (64)	31 (54)	-	-	5 (16)	-
Double	*Pliln1*-Cas<br>*Kcnq4*-Cas	100	61 (61)	44 (72)	-	-	2 (5)	-
Triple	*Plin1*-Cas<br>*Kcnq4*-Cas<br>*Fgfr3*-Cas	99	54 (55)	34 (63)	-	-	3 (9)	-

Supplementary Fig. 9 Embryonic development rates following the application of CRISPR-VIM method. a, b, Analysis of embryonic development rates and offspring ratios following the CRISPR-VIM method in (**a**) zygotes and (**b**) IVF. + indicates 10% and ++ indicates 20% VLP treatment of the total medium volume. **c, d,** Evaluation of developmental rates and offspring proportions in mouse embryos resulting from (**c**) the knock-in approach at the *Kcnq4* gene and (**d**) multi-gene targeting.

Cargo protein	Target sgRNA	VLPs/µl
Cas9	*Clcn7*	2.60×10^{8}
	Ddc	3.61×10^{8}
	Dnmt1	2.24×10^{8}
	Fgfr3	3.59×10^{8}
	Fgfr3+Kcnq4	3.96×10^{8}
	Gata3	3.50×10^{8}
	Gjb2	2.96×10^{8}
	Hcrt	1.90×10^{8}
	Igf2	2.01×10^{8}
	Kcnq4	3.94×10^{8}
	Kcnq4-sgRNA1	5.10×10^{10}
	Kcnq4-sgRNA2	1.45×10^{10}
	Kcnq4+Plin1	2.57×10^{8}
	Lep	2.43×10^{8}
	Nanog	1.54×10^{8}
	Pdgfra	2.26×10^{8}
	Plin1	3.00×10^{8}
	Tyr-Ex1	2.76×10^{8}
	Tyr-Ex4	1.76×10^{8}
	HBB	1.84×10^{8}
	HEK3	2.47×10^{8}
	TTN	2.91×10^{8}
ABE8e	*Dnmt1*	2.82×10^{8}
	Gata3	3.07×10^{8}
	Gata3+Kcnq4	1.25×10^{8}
	Igf2	2.27×10^{8}
	Kcnq4	1.64×10^{8}
	Lep	3.02×10^{8}
	Nanog	2.64×10^{8}
	Pdgfra	1.97×10^{8}
	Plin1	1.78×10^{8}
	Tyr-Ex4	2.27×10^{8}
	HBB	1.42×10^{8}
	HEK3	2.27×10^{8}
	TTN	1.42×10^{8}
CBE(WT)	*Cftr*	4.41×10^{8}
	Dip2a	2.69×10^{8}
	Dnmt1	2.71×10^{8}
	F9	5.09×10^{8}
	Gjb2	4.63×10^{8}
	Hpd	5.52×10^{8}
	Kcnq4	4.13×10^{8}
	Rpe65	1.84×10^{8}
	Tyr-Ex4	4.03×10^{8}
	ADAMTS4	3.90×10^{8}
	CCR5	4.65×10^{8}
	EIF3D	4.14×10^{8}
	EMX1	4.93×10^{8}
	HEK3	6.34×10^{8}
	MYOCD	4.08×10^{8}
	RNF2	3.51×10^{8}
CBE(OPT)	*Cftr*	2.43×10^{8}
	Dip2a	1.54×10^{8}
	Dnmt1	4.62×10^{8}
	F9	3.00×10^{8}
	Gjb2	3.61×10^{8}
	Hpd	4.30×10^{8}
	Kcnq4	1.76×10^{8}
	Rpe65	1.84×10^{8}
	Tyr-Ex4	2.47×10^{8}
	ADAMTS4	2.13×10^{8}
	CCR5	2.15×10^{8}
	EIF3D	1.31×10^{8}
	EMX1	1.65×10^{8}
	HEK3	4.47×10^{8}
	MYOCD	3.25×10^{8}
	RNF2	2.73×10^{8}

Supplementary Fig. 10 Quantification of VLPs for all experimental targets using P30 ELISA. P30 ELISA for quantifying VLP concentrations across all experimental targets.

Supplementary Table 1. Sequences of single guide RNAs targeting human and mouse genomes.

Target	Sequence with PAM (5' to 3')
ADAMTS4	TGGCACCATCAATGGAGATCCGG
CCR5	TGACATCAATTATTATACATCGG
EIF3D	AGACGACCCTGTCATCCGCAAGG
EMX1	GAGTCCGAGCAGAAGAAGAAGGG
HBB	TCAGAAAGTGGTGGCTGGTGTGG
HEK3	GGCCCAGACTGAGCACGTGATGG
MYOCD	ACTTCAACAAAGAAGGACCCAGG
RNF2	GTCATCTTAGTCATTACCTGAGG
TTN	GGTACTTCAGCAGCCTTCACTGG
Cftr	CTTATATCTGTACTCATCATAGG
Clcn7	GGGGGATCTTCACCCCATTGAGG
Ddc	AATGAAAGCAGAGCTGCTTCAGG
Dip2a	CATTCAAGGTATGGGCAGCGTGG
Dnmt1	AACAGCTCTGAACGAGACCCCGG
F9	CTACTCAGTACCGAATGTGCAGG
Fgfr3	GAGGCTGGCAGCGTGTACGCAGG
Gata3	GTCACCGCCATGGGTTAGAGAGG
Gjb2	ACTTCCCCATCTCTCACATCCGG
Hcrt	TCAGGACAAGGATAGAAGATGGG
Hpd	CAACCCAGAAGGTCACCGAGTGG
Igf2	TATTGGAAGAACTTGCCCACGGG
Kcnq4	CCTATGCCGACTCGCTCTGGTGG
Kcnq4-1	CTGGGTTGAAGCCTGAAAGATGG
Kcnq4-2	GATACAAACAGGTTGCCCATAGG
Lep	CCAGCAGATGGAGGAGGTCTCGG
Nanog	TATGAGACTTACGCAACATCTGG
Pdgfra	ACCCGGAGCGTGTCAGTTACAGG
Plin1	TGCCTATGAGAAGGGTGTACAGG
Rpe65	GTCTCCTCCGATGTGGGCCAGGG
Tyr-Ex1	GGGTGGATGACCGTGAGTCCTGG
Tyr-Ex4	CCATAACAGAGACTCTTACATGG

Supplementary Table 2. Primer sequences for on-target gene editing analysis.

	1st PCR		2nd PCR	
Target	Forward (5' to 3')	Reverse (5' to 3')	Forward (5' to 3')	Reverse (5' to 3')
ADAMTS4	GGAGATCGTGTTTCCAGAGAAG	GAACGGCCAGAAGTGTAAGT	ACACTCTTTCCCTACACGACGCTCTTCCGATCTGGCTGACAGTGCAGTACC	GTGACTGGAGTTCAGACGTGTGCTCTTCCGATCTACTCTTCCGGCGTAGGAT
CCR5	AGAGCCAAGCTCTCCATCTA	GAAGATTCCAGAGAAGAAGCCTATAA	ACACTCTTTCCCTACACGACGCTCTTCCGATCTCATTCATGGAGGGCAACTAAATAC	GTGACTGGAGTTCAGACGTGTGCTCTTCCGATCTAAGATGAACACCAGTGAGTAGAG
EIF3D	GTTGTGCTTGCTGTGTTCTATG	GCTTCAGATGCTTTCACCTTTG	ACACTCTTTCCCTACACGACGCTCTTCCGATCTATGTCTTTGCCTGCTCTTCC	GTGACTGGAGTTCAGACGTGTGCTCTTCCGATCTAAGCCGACAGCATTCCTAAC
EMX1	CAGCTCTGTGACCCTTTGTT	TGCTTGTCCCTCTGTCAATG	ACACTCTTTCCCTACACGACGCTCTTCCGATCTCTGGCCCAGGTGAAGGT	GTGACTGGAGTTCAGACGTGTGCTCTTCCGATCTTCGTGGGTTTGTGGTTGC
HBB	CTCTTTCTTTCAGGGCAATAATGATAC	GGCAGAATCCAGATGCTCAA	ACACTCTTTCCCTACACGACGCTCTTCCGATCTACCTCTTATCTTCCTCCCACA	GTGACTGGAGTTCAGACGTGTGCTCTTCCGATCTAGTTGGACTTAGGGAACAAAGG
HEK3	GTTGAGCTCGACCCTGAAG	GGCATGAGAAACCTTGGAGA	ACACTCTTTCCCTACACGACGCTCTTCCGATCTCCAAACTTGTCAACCAGTATCC	GTGACTGGAGTTCAGACGTGTGCTCTTCCGATCTGCATGCATTTGTAGGCTTGA
MYOCD	CAGAGTGTAGAGAGAGAGAGCA	CCCTGAGAACAAATCCACTACA	ACACTCTTTCCCTACACGACGCTCTTCCGATCTTGTAATGATATTTAGCCCAACTCAC	GTGACTGGAGTTCAGACGTGTGCTCTTCCGATCTTTGGCTTAAACTAAATGACCTCTG
RNF2	CATAACCTGATCACCTCCCAAA	AGGACTTGCCCAACTTTCTAC	ACACTCTTTCCCTACACGACGCTCTTCCGATCTTGCAGACAAACGGAACTCAA	GTGACTGGAGTTCAGACGTGTGCTCTTCCGATCTGCCAACATACAGAAGTCAGGAA
TTN	CTGCCTGCCAGAAACTACA	TCTGAACTCCAGGACTGACT	ACACTCTTTCCCTACACGACGCTCTTCCGATCTTTTGTCGGAGAAGACTCCATTC	GTGACTGGAGTTCAGACGTGTGCTCTTCCGATCTCTGTTCACCTTTACCTTTCCTTTC
Clcn7	GCAGAACCTGCTCTGTGTATAG	CCAGATGTGGAGAAGGATCAAG	ACACTCTTTCCCTACACGACGCTCTTCCGATCTCCCTTACTTTACCTGCTCATTGG	GTGACTGGAGTTCAGACGTGTGCTCTTCCGATCTTGGAGAAGGATCAAGCAAAGAC
Ddc	GTCTACCGTCTCTAGGTGTTACT	GCTTTGGTCAGTGCTGTTTG	ACACTCTTTCCCTACACGACGCTCTTCCGATCTCTCGCACTGTGGAGTCTG	GTGACTGGAGTTCAGACGTGTGCTCTTCCGATCTCACAGCCACTCAGCACTAA
Dip2a	CTGGGAAGGCAGAGAAGAAA	CCCAAGACCTATCCCATGTTAG	ACACTCTTTCCCTACACGACGCTCTTCCGATCTTCCCATTGTTGTAGGTGACATC	GTGACTGGAGTTCAGACGTGTGCTCTTCCGATCTCCCATGTTAGCAAGGGTTCTAC
Dnmt1	TGGCAAGCAAACCAGAGT	TGGCGCCAACAGCTAAG	ACACTCTTTCCCTACACGACGCTCTTCCGATCTCTCCCTTCGGGCATAGCAT	GTGACTGGAGTTCAGACGTGTGCTCTTCCGATCTCTCGGGCTGGAGCTGTT
F9	CAAAGATACACCGAGGGAGATG	CCACAAGCCCTGTAAATGTAGT	ACACTCTTTCCCTACACGACGCTCTTCCGATCTAACACCGTCATGGCAGAAT	GTGACTGGAGTTCAGACGTGTGCTCTTCCGATCTGACGTGCTTCCTGCTACAATA
Fgfr3	CTTGGTGTGGAAACATGAATCT	CTAGCTGCTCGGACTTCTATAC	ACACTCTTTCCCTACACGACGCTCTTCCGATCTTCTTCTCCAAGTATCCCAGGT	GTGACTGGAGTTCAGACGTGTGCTCTTCCGATCTGCCACCACCAGGATGAAG
Gata3	ATCCCTGAGCCACATCTCT	CCGGATTCAGTGGTTGGAAT	ACACTCTTTCCCTACACGACGCTCTTCCGATCTCACTCCAGCCACATGCT	GTGACTGGAGTTCAGACGTGTGCTCTTCCGATCTGTCGCTTTCGGGCTTCA
Gjb2	CTCCACCAGCATTGGAAAGA	AAAGATGACCCGGAAGAAGATG	ACACTCTTTCCCTACACGACGCTCTTCCGATCTTATGCTACGACCACCACTTC	GTGACTGGAGTTCAGACGTGTGCTCTTCCGATCTGATACGGACCTTCTGGGTTT
Hcrt	GTATCCTGACTCTGGGAAAGC	AGTCACACCAACAGAGAATCG	ACACTCTTTCCCTACACGACGCTCTTCCGATCTTAGAGCCACATCCCTGCTC	GTGACTGGAGTTCAGACGTGTGCTCTTCCGATCTACACCAACAGAGAATCGTCTTTAT
Hpd	GTGGTCACCCATACTGTTCTC	CAGAGGTTCAAAGCCCATCT	ACACTCTTTCCCTACACGACGCTCTTCCGATCTTTTGGGCTTGAGGCTGA	GTGACTGGAGTTCAGACGTGTGCTCTTCCGATCTGAGGCCCTTTGGCTTGT
Igf2	GTGAGACAAAGAGACCACTCAC	CTCAAGAGGAGGTCACAGATTG	ACACTCTTTCCCTACACGACGCTCTTCCGATCTTAGATGGGAGCTCAGGCTAA	GTGACTGGAGTTCAGACGTGTGCTCTTCCGATCTGACGTTTGGCCTCTCTGAA
Kcnq4	GCCTGAAAGATGGAGCAAGA	CCACAGCCTTAGTGTGAAGAG	ACACTCTTTCCCTACACGACGCTCTTCCGATCTTGATCACCGCCTG	GTGACTGGAGTTCAGACGTGTGCTCTTCCGATCTTAGGAAGACA

			GTACAT	GAACCTGGGAAG
Lep	TGGCTCGGAATGAAC AGAAA	CTTTGGATGGGTGGTC TACAG	ACACTCTTTCCCTACACGACGCT CTTCCGATCTCCCATTCTGAGTT TGTCCAAGAT	GTGACTGGAGTTCAGACGTGTG CTCTTCCGATCTTTCCAGGACGC CATCCA
Nanog	AGGACTTTCTGCAGCC TTAC	TGGTGGCTCACAACC ATAC	ACACTCTTTCCCTACACGACGCT CTTCCGATCTACTAGGGAAAGC CATGCG	GTGACTGGAGTTCAGACGTGTG CTCTTCCGATCTGTGTCTGAAGA CAGCTACAGTG
Pdgfra	GTTCCAGTAGTTCCAC CTTCAT	ACTCTCTTGTCCATCC ATTTCC	ACACTCTTTCCCTACACGACGCT CTTCCGATCTCATCGACATGATG GATGACATTG	GTGACTGGAGTTCAGACGTGTG CTCTTCCGATCTGCCTCGGGAAC TTTCTCTC
Plin1	GGAAAGGACCACAGT TCTATCA	CACATACCCACAAGCC TCTATC	ACACTCTTTCCCTACACGACGCT CTTCCGATCTTTCTTTGCTGACA GGAGCAG	GTGACTGGAGTTCAGACGTGTG CTCTTCCGATCTAGTCTATGAGG ACTCACACTGG
Rpe65	TCCTGACACTTATCCC TGTATCT	GTCATGGACTTACCTT CTGTGG	ACACTCTTTCCCTACACGACGCT CTTCCGATCTGTTCTGTCTATAG GTAAGCTGACAA	GTGACTGGAGTTCAGACGTGTG CTCTTCCGATCTGCCCTCCTTGA AGTCAAACT
Tyr-Ex1	AGTGCTATTCAAACCA TCCAGTAA	ACCTTTGCTATTTACC AACAATGC	TCAGAATCTAGATGTTTCATGAC CT	GTGAACCAATCAGTCCTTGTTTA TAG
Tyr-Ex4	CCTCACACTACTTCTG ATGAATGA	CCCACATTGCATTCCA TAGTTC	ACACTCTTTCCCTACACGACGCT CTTCCGATCTTCCTGACTCTGAG TAACCCTTC	GTGACTGGAGTTCAGACGTGTG CTCTTCCGATCTGACTCTTGGAG GTAGCTGTAGT
Tyr-Ex1 (1434 bp)	TCAGAATCTAGATGTT TCATGACCT	GTGAACCAATCAGTCC TTGTTTATAG		
Kcnq4 (680 bp)	AACCTGGGCTTCTGTT CTTC	CTTGGTTCCTCTCACC TTTCTC		
Kcnq4 (985 bp)	CCCTTCTGTGTCATCG GTATAAA	CCACACGGGAAAGGG ATAAA		
Kcnq4 (3929 bp)	AACACTTCCTTGGGAC CTTAAA	ACCAGAGCAGGGAGA TATAGAA		
Kcnq4 (2046bp)	TTTATCCCTTTCCCGTG TGG	CCTTTGGGTGCCTCTT GT		

122

123

Supplementary Table 3. Sequences of off-target candidate sites for *Tyr* and *Plin1*.

Target	Sequence with PAM (5' to 3')
Tyr-OT1	ttATAACAGAGACTCTTACtGGG
Tyr-OT2	CgATtACAGAGACTCcTACAAGG
Tyr-OT3	CCATAACtGAGACTCTgcCAAGG
Tyr-OT4	tCcaAACAGAGACTCTTACAAGG
Tyr-OT5	aCATAgCAGgGACTCTTACACGG
Tyr-OT6	CgATcACAGAaACTCTTACAAGG
Tyr-OT7	CCAcAACAcAGACTCTTAaAAGG
Tyr-OT8	tCtTAACAGAGACTCTTAaAAGG
Tyr-OT9	CaATAACAGAGgtTCTTACAAGG
Plin1-OT1	TGCCTcTGAGAgGtGTGTACTGG
Plin1-OT2	TGCCTcTGAGAgGtGTGTACTGG
Plin1-OT3	TGCCTcTGAGAAcaGTGTACCGG
Plin1-OT4	TGCCTcTGAaAAGGGTGTgCTGG
Plin1-OT5	TGCCTAaGAGAAGGGTGaAtaGGG
Plin1-OT6	TGCCggTGAGAAGGGTGTAgTGG
Plin1-OT7	ctCCTATGAGAAGGtTGTACGGG
Plin1-OT8	TGgCTcTtAGAAGGGTGTACTGG
Plin1-OT9	TGCCTtTGAGAAGGaTGcACAGG
Plin1-OT10	TGCaTATcAGAAtGGTGTACAGG

Supplementary Table 4. Primer sequences for analyzing off-target effects of *Tyr* and *Plin1*.

	1st PCR		2nd PCR	
Target	Forward (5' to 3')	Reverse (5' to 3')	Forward (5' to 3')	Reverse (5' to 3')
Tyr-OT1	GGGTGTCATTCCTCAGTTCTTT	CCCACAAGTAATTCTCTCCTGATAC	ACACTCTTTCCCTACACGACGCTCTTCCGATCTTTCTGGGTGTGGAATTCAGG	GTGACTGGAGTTCAGACGTGTGCTCTTCCGATCTACCAGAGAAAGCTGATGGTAAG
Tyr-OT2	CCAAAGTATGTACACAGTTGCTT	TCAGCAGGAATTCTGGTTCTC	ACACTCTTTCCCTACACGACGCTCTTCCGATCTCTGACTTGTTGGAAAGAGCAAAG	GTGACTGGAGTTCAGACGTGTGCTCTTCCGATCTGTTTGAAGGTTACCCGCTACA
Tyr-OT3	ACAACAGGCACAGGATTAGG	GCTGCAGAGACTCTTGATTGA	ACACTCTTTCCCTACACGACGCTCTTCCGATCTCAACAGGCACAGGATTAGGA	GTGACTGGAGTTCAGACGTGTGCTCTTCCGATCTCCTGCTACGTAGATGGCATAG
Tyr-OT4	GGGAAGTAATCAGTCGCAGAG	GTCTGGCAAGCAAACCTAGA	ACACTCTTTCCCTACACGACGCTCTTCCGATCTCCCAACAAACTGTGGGAAATAAA	GTGACTGGAGTTCAGACGTGTGCTCTTCCGATCTTGTAAGAGCATCGAGCCATTT
Tyr-OT5	TAACAGAAGCCTCTTGTTCCC	CAGCCATACCTCTGGTCAAA	ACACTCTTTCCCTACACGACGCTCTTCCGATCTTAAGGTCAGCTGGGTCCATTAG	GTGACTGGAGTTCAGACGTGTGCTCTTCCGATCTAGAAAGAGCAGAGGGAGAGAA
Tyr-OT6	GCATTGGCAGCAGGAATAAAT	GCGGTACTACTTGAGAAGGATTAG	ACACTCTTTCCCTACACGACGCTCTTCCGATCTCTATTGCTGTGAAGAGATGCC	GTGACTGGAGTTCAGACGTGTGCTCTTCCGATCTGGAAGTATGACCTTGCTGGAG
Tyr-OT7	CTTGTGGTGCTGGAGATCAA	CGAAGCTCTTTAGAGGAGCATAA	ACACTCTTTCCCTACACGACGCTCTTCCGATCTGTGGTGCTGGAGATCAAACT	GTGACTGGAGTTCAGACGTGTGCTCTTCCGATCTGCTTCTCTACTTCCCAAGTTCTC
Tyr-OT8	TGAGGAGTCCCAGTTCTGA	GGCTGGATTTGTGGGTAGAT	ACACTCTTTCCCTACACGACGCTCTTCCGATCTTGCTCCTGCCACATAATAGTC	GTGACTGGAGTTCAGACGTGTGCTCTTCCGATCTGGCTGGATTTGTGGGTAGAT
Tyr-OT9	GCATGAATGAGGCTTTGTTCTC	TCGGAGGACCCTGGATTT	ACACTCTTTCCCTACACGACGCTCTTCCGATCTGTGTAGGTGCATGGAGGTATTAG	GTGACTGGAGTTCAGACGTGTGCTCTTCCGATCTGGCTTACAGATGGGTGTCTTT
Plin1-OT1	CCCATTGAGGCTGCAGTTAT	CATGGACAGGTGGTGTAGAAAG	ACACTCTTTCCCTACACGACGCTCTTCCGATCTACTGTGTTCCAGCCCTTTG	GTGACTGGAGTTCAGACGTGTGCTCTTCCGATCTCTGCACACTACTGACCATGAC
Plin1-OT2	CTTATCCTTCCAGGTCACTG	CATGGACAGGTGGTGTAGAAA	ACACTCTTTCCCTACACGACGCTCTTCCGATCTCTCTTGCTAGACCTGTCATGAGG	GTGACTGGAGTTCAGACGTGTGCTCTTCCGATCTCCCAGAAAGTATGGGTGTCAAT
Plin1-OT3	GCCAAAGCCCAAATGGTTAT	GGACTCGTTTGTCCAGTATGT	ACACTCTTTCCCTACACGACGCTCTTCCGATCTGTGCCATGTCAATAGCAGATAAG	GTGACTGGAGTTCAGACGTGTGCTCTTCCGATCTGGGATGGGTAAGCTAGAAATGT
Plin1-OT4	GAATGTGGGTAGGAGGACTTAAC	CTAATTGGGAAGTCTCGAAGGG	ACACTCTTTCCCTACACGACGCTCTTCCGATCTTGGAGGTGAGAACCATCTCT	GTGACTGGAGTTCAGACGTGTGCTCTTCCGATCTACAAAGCAATGAGGTGCCTA
Plin1-OT5	CTTCGTCACCCTGCTCATTTA	CCTAGAACTTGCCTCTCTTATGG	ACACTCTTTCCCTACACGACGCTCTTCCGATCTCGCCTCAAGGCAGTTTCA	GTGACTGGAGTTCAGACGTGTGCTCTTCCGATCTCAGAGTTCCTGAGTCTGGTAAATAA
Plin1-OT6	ACTGAGACCCAGAGGGAATAA	GTCAGGATGACACCCATCTTC	ACACTCTTTCCCTACACGACGCTCTTCCGATCTGGCTGTAGAAAGGCAGGTATTT	GTGACTGGAGTTCAGACGTGTGCTCTTCCGATCTACCCTGTACCACAGGTCTC
Plin1-OT7	GTGTGATGTGAGGGTCAGAAA	GGAGTAAGAAGGGCCGTAAAG	ACACTCTTTCCCTACACGACGCTCTTCCGATCTGAGACAGACATGTGACCAAGAG	GTGACTGGAGTTCAGACGTGTGCTCTTCCGATCTTGCAAGTATAGGGAGGACAGA
Plin1-OT8	GTCAACCTCTGGTACTGTGAAA	AACTCAAGCAGGTCCGAAAG	ACACTCTTTCCCTACACGACGCTCTTCCGATCTAACAATAGGGACTTGCCTTGA	GTGACTGGAGTTCAGACGTGTGCTCTTCCGATCTCTGAAGCTCCTTTCTCTGTGAT
Plin1-OT9	AACCATCACGTGGGAGAATG	CCTGTGGCTCAGAAGTCATTTA	ACACTCTTTCCCTACACGACGCTCTTCCGATCTCCGGGAGGAAGAATCAAATGTA	GTGACTGGAGTTCAGACGTGTGCTCTTCCGATCTCCTGTGGCTCAGAAGTCATTTA
Plin1-OT10	CTGAACAAGGGCAACACTAGA	CCAATACCAGTTGACCCAGAA	ACACTCTTTCCCTACACGACGCTCTTCCGATCTCCATCCTACACAAAGACCTACA	GTGACTGGAGTTCAGACGTGTGCTCTTCCGATCTGGACCAACAGAACAAACGATAC
