# Supplementary material for: An innovative approach using CRISPR-ribonucleoprotein packaged in virus-like particles to generate genetically engineered mouse models
Source: Nat Commun. 2025 Apr 11;16:3451. doi: 10.1038/s41467-025-58364-7 (PMC11992299; doi:10.1038/s41467-025-58364-7)
Supplement: Supplementary file 2 — Reporting Summary [file 41467_2025_58364_MOESM2_ESM.pdf]

Corresponding author(s): NCOMMS-24-09087

Last updated by author(s): Mar 18, 2025

Reporting Summary

Nature Portfolio wishes to improve the reproducibility of the work that we publish. This form provides structure for consistency and transparency in reporting. For further information on Nature Portfolio policies, see our Editorial Policies and the Editorial Policy Checklist.

Statistics

For all statistical analyses, confirm that the following items are present in the figure legend, table legend, main text, or Methods section.

n/a	Confirmed	
☐	☒	The exact sample size (*n*) for each experimental group/condition, given as a discrete number and unit of measurement
☐	☒	A statement on whether measurements were taken from distinct samples or whether the same sample was measured repeatedly
☐	☒	The statistical test(s) used AND whether they are one- or two-sided<br>*Only common tests should be described solely by name; describe more complex techniques in the Methods section.*
☒	☐	A description of all covariates tested
☒	☐	A description of any assumptions or corrections, such as tests of normality and adjustment for multiple comparisons
☐	☒	A full description of the statistical parameters including central tendency (e.g. means) or other basic estimates (e.g. regression coefficient) AND variation (e.g. standard deviation) or associated estimates of uncertainty (e.g. confidence intervals)
☒	☐	For null hypothesis testing, the test statistic (e.g. *F*, *t*, *r*) with confidence intervals, effect sizes, degrees of freedom and *P* value noted<br>*Give P values as exact values whenever suitable.*
☒	☐	For Bayesian analysis, information on the choice of priors and Markov chain Monte Carlo settings
☒	☐	For hierarchical and complex designs, identification of the appropriate level for tests and full reporting of outcomes
☒	☐	Estimates of effect sizes (e.g. Cohen's *d*, Pearson's *r*), indicating how they were calculated

Our web collection on statistics for biologists contains articles on many of the points above.

Software and code

Policy information about availability of computer code

Data collection	Illumina MiSeq and MiniSeq platform were used to collect the targeted deep sequencing data. Image Lab™ Software was used for capturing Immunoblot images. CaseViewer (Version 2.4.0.119028 by 3DHISTECH Ltd) was used to acquire Hematoxylin and eosin (H&E) staining image and Immunohistochemistry images. Zeiss Zen software (Zen 2.6 Blue edition) was used to obtain Immunofluorescence images.
Data analysis	CRISPR RGEN Tools and EUN program were used to quantifying edited reads and to search potential off-target sites. Microsoft Excel and Graphpad prism were used to analyze the targeted deep sequencing data.

For manuscripts utilizing custom algorithms or software that are central to the research but not yet described in published literature, software must be made available to editors and reviewers. We strongly encourage code deposition in a community repository (e.g. GitHub). See the Nature Portfolio guidelines for submitting code & software for further information.

Data

Policy information about availability of data

All manuscripts must include a data availability statement. This statement should provide the following information, where applicable:

- Accession codes, unique identifiers, or web links for publicly available datasets
- A description of any restrictions on data availability
- For clinical datasets or third party data, please ensure that the statement adheres to our policy

The authors declare that all data supporting the findings of this study are available in the article and its supplementary Information files. All other data supporting the findings of this study are available from the corresponding author on request. Targeted deep sequencing data files will be uploaded at the National Center for

Biotechnology Information (NCBI) before publication.

Research involving human participants, their data, or biological material

Policy information about studies with human participants or human data. See also policy information about sex, gender (identity/presentation), and sexual orientation and race, ethnicity and racism.

Reporting on sex and gender	N/A
Reporting on race, ethnicity, or other socially relevant groupings	N/A
Population characteristics	N/A
Recruitment	N/A
Ethics oversight	N/A

Note that full information on the approval of the study protocol must also be provided in the manuscript.

Field-specific reporting

Please select the one below that is the best fit for your research. If you are not sure, read the appropriate sections before making your selection.

- [x] Life sciences
- [ ] Behavioural & social sciences
- [ ] Ecological, evolutionary & environmental sciences

For a reference copy of the document with all sections, see nature.com/documents/nr-reporting-summary-flat.pdf

Life sciences study design

All studies must disclose on these points even when the disclosure is negative.

Sample size	Sample sizes for cell experiments were determined based on literature precedence for genome editing experiments.The sample size for Plin1 analysis was determined based on experimental feasibility, as the generation of transgenic mice required a prolonged breeding process. Previous studies (Kim et al. Nat Methods, 2024) have shown that similar sample sizes (n= 2-3) were sufficient to draw reliable conclusions.
Data exclusions	No data was excluded.
Replication	All attempts at replication were successful.
Randomization	Obtained mouse oocytes and zygotes were allocated randomly to the different VLP treatment groups
Blinding	Blinding was not used.

Reporting for specific materials, systems and methods

We require information from authors about some types of materials, experimental systems and methods used in many studies. Here, indicate whether each material, system or method listed is relevant to your study. If you are not sure if a list item applies to your research, read the appropriate section before selecting a response.

Materials & experimental systems

n/a	Involved in the study	
	x	Antibodies
	x	Eukaryotic cell lines
x		Palaeontology and archaeology
	x	Animals and other organisms
x		Clinical data
x		Dual use research of concern
x		Plants

Methods

n/a	Involved in the study	
x		ChIP-seq
x		Flow cytometry
x		MRI-based neuroimaging

Antibodies

Antibodies used	Anti-MuLV p30 Polyclonal Antibody and Secondary Antibody, HRP Conjugate in MuLV Core Antigen ELISA kit (Cell Biolabs; VPK-156); Biotin-labeled antibody and HRP-Streptavidin conjugate (SABC) in FLAG-Tag (DYKDDDDK-Tag protein) ELISA kit (Finetest; EU2607); Anti-Perilipin 1(Invitrogen, PA1-1051); Anti-α-Tubulin(Abbkine, A01080);Mouse anti-rabbit IgG-HRP(Santa Cruz Biotechnology,

sc-2357);M-IgGκ BP-HRP(Santa Cruz Biotechnology, sc-516102);Anti-F4/80(Cell signaling, 70076);Goat anti-rabbit IgG cross-adsorbed secondary antibody(Invitrogen, A21245)

Validation	Antibody was validated by antibody suppliers.

Eukaryotic cell lines

Policy information about cell lines and Sex and Gender in Research

Cell line source(s)	HEK293T, APRE19,Neuro-2a and mouse embryonic stem cells (C57BL/6;SCRC-1002)were obtained from ATCC. Gesicle producer 293T was purchased from TaKaRa Bio.
Authentication	Used cell lines were not authenticated in the laboratory after purchase.
Mycoplasma contamination	Mycoplasma were not detected from used cell lines.
Commonly misidentified lines (See ICLAC register)	Misidentified cell lines were not used.

Animals and other research organisms

Policy information about studies involving animals; ARRIVE guidelines recommended for reporting animal research, and Sex and Gender in Research

Laboratory animals	Female (5-8w), male (10-26w) C57BL/6 and ICR (8-20w) mice were from DBL Co., Ltd.(Korea) and Orient Bio Inc. (Korea).
Wild animals	No wild animals were used.
Reporting on sex	Sex-based analysis was not performed.
Field-collected samples	No field-collected samples were used.
Ethics oversight	All animal procedures were conducted with guidelines from Institutional Animal Care and Use Committee of Seoul National University (SNU-220930-2-1, SNU-230813-1-1) and Korea University (KOREA-2022-0105-C2, KOREA-2022-0105).

Note that full information on the approval of the study protocol must also be provided in the manuscript.

Plants

Seed stocks	N/A
Novel plant genotypes	N/A
Authentication	N/A
